# RNA mutagenesis yields highly diverse mRNA libraries for *in vitro *protein evolution

**DOI:** 10.1186/1472-6750-7-18

**Published:** 2007-04-11

**Authors:** George Kopsidas, Rachael K Carman, Emma L Stutt, Anna Raicevic, Anthony S Roberts, Mary-Anne V Siomos, Nada Dobric, Luisa Pontes-Braz, Greg Coia

**Affiliations:** 1 EvoGenix Ltd., 343 Royal Parade, Parkville, Melbourne 3052, Australia; 2 CSIRO, Molecular and Health Technologies, 343 Royal Parade, Parkville, Melbourne 3052, Australia

## Abstract

**Background:**

In protein drug development, *in vitro *molecular optimization or protein maturation can be used to modify protein properties. One basic approach to protein maturation is the introduction of random DNA mutations into the target gene sequence to produce a library of variants that can be screened for the preferred protein properties. Unfortunately, the capability of this approach has been restricted by deficiencies in the methods currently available for random DNA mutagenesis and library generation. Current DNA based methodologies generally suffer from nucleotide substitution bias that preferentially mutate particular base pairs or show significant bias with respect to transitions or transversions. In this report, we describe a novel RNA-based random mutagenesis strategy that utilizes Qβ replicase to manufacture complex mRNA libraries with a mutational spectrum that is close to the ideal.

**Results:**

We show that Qβ replicase generates all possible base substitutions with an equivalent preference for mutating A/T or G/C bases and with no significant bias for transitions over transversions. To demonstrate the high diversity that can be sampled from a Qβ replicase-generated mRNA library, the approach was used to evolve the binding affinity of a single domain V_NAR _shark antibody fragment (12Y-2) against malarial apical membrane antigen-1 (AMA-1) via ribosome display. The binding constant (K_D_) of 12Y-2 was increased by 22-fold following two consecutive but discrete rounds of mutagenesis and selection. The mutagenesis method was also used to alter the substrate specificity of β-lactamase which does not significantly hydrolyse the antibiotic cefotaxime. Two cycles of RNA mutagenesis and selection on increasing concentrations of cefotaxime resulted in mutants with a minimum 10,000-fold increase in resistance, an outcome achieved faster and with fewer overall mutations than in comparable studies using other mutagenesis strategies.

**Conclusion:**

The RNA based approach outlined here is rapid and simple to perform and generates large, highly diverse populations of proteins, each differing by only one or two amino acids from the parent protein. The practical implications of our results are that suitable improved protein candidates can be recovered from *in vitro *protein evolution approaches using significantly fewer rounds of mutagenesis and selection, and with little or no collateral damage to the protein or its mRNA.

## Background

There is a growing demand by the pharmaceutical and medical industries for protein molecules, including antibodies, of diagnostic and therapeutic efficacy, as well as a perpetual need in the production and manufacturing industries for improved biocatalysts. These demands have directed the innovation of a number of sophisticated and complex methods for the *in vitro *evolution and optimization of proteins [1]. One fundamental approach to this process is the introduction of random mutations into a known nucleotide sequence to produce a library of variants. These variants are subsequently translated to produce modified proteins that are accordingly screened for chosen properties.

The potential of this approach has been limited by deficiencies in the methods currently available for random mutagenesis and library generation [2]. Current methods exclusively target DNA, and include error-prone PCR (EP-PCR) [3], the incorporation of triphosphate derivatives of nucleoside analogues with *Taq *or other DNA polymerases [4] and novel error-prone DNA polymerases or polymerase blends [5,6]. Unfortunately, DNA-based mutagenesis systems generally suffer from a nucleotide incorporation bias that favors transitions over transversions and/or results in a skewed preference for mutations at either A/T or G/C pairs [7,8]. Without doubt, base substitution bias will diverge the distribution of mutations from a Poisson distribution, effectively diminishing the functional size of a randomly mutated gene library available for subsequent screening [9]. In essence, any nucleotide bias reduces the probability for generating specific amino acid substitutions that may be required at key positions along the protein, dramatically reducing the potential for recovering protein variants with a desired set of properties. Directed protein evolution using powerful selection strategies such as ribosome display (described below) are more likely to identify improved variants when a library is maximally diverse which would be the case when all variants in a library are equally probable [10].

We have exploited Qβ bacteriophage RNA replicase, an error-prone RNA-dependent RNA polymerase, and its ability to amplify and mutate RNA very rapidly, to develop an *in vitro *mutagenesis strategy targeting mRNA. We have found that base substitutions at the RNA level are made with very little bias for the incorporation of particular bases, approaching what can be considered as ideal random mutagenesis. The result is the generation of random mRNA libraries carrying very high diversity.

To verify that Qβ replicase manufactured variant mRNA libraries can be highly effective tools for *in vitro *protein evolution, two basic demonstrations are presented here. First, Qβ replicase mutagenesis in combination with a simple functional assay was used to alter the substrate specificity of β-lactamase for the antibiotic cefotaxime. And second, Qβ mRNA mutagenesis coupled to ribosome display was used to enhance the affinity of a single domain antibody fragment (V_NAR_) to its antigen. Ribosome display is an *in vitro *display and selection strategy that couples the newly translated protein to the ribosome complex, which in turn, remains tethered to the mRNA message due to the absence of a stop codon on the mRNA (effectively linking phenotype to genotype). An mRNA library can be translated *in vitro *and the ribosome-protein-mRNA complexes can be subsequently screened (panned) for binding towards the appropriate molecule and non-specific or weaker binding complexes removed by extensive washing. [11,12]. The mRNA is eluted from the remaining (bound) ribosome complexes and amplified with RT-PCR. Ribosome display, in particular, is ideal for the effective screening of large mRNA libraries, with the number of variants that can be screened limited only by the total number of ribosomes in solution (estimated to be up to 10^14^/ml typically found with *in vitro *eukaryotic reticulocyte lysate systems) and the total amount of mRNA that can be added to the translation mix [13].

## Results

### Constructing the Qβ replicase mutagenesis and ribosome display vectors

**Figure 1:**
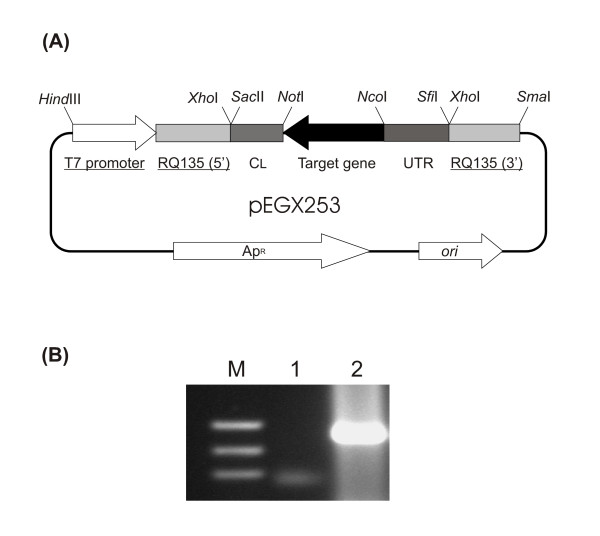
**Qβ replicase mutagenesis/ribosome display vector and typical Qβ replicase amplification reaction**. **(A): **Schematic representation of plasmid pEGX253. Base plasmid pEGX216 (elements depicted with underline) comprised of a MCS site inserted into a modified RQ 135_-1_(-) sequence which was then used to add the required elements to construct pEGX253. Target gene sequence (12Y-2) was cloned into the *Nco*I and *Not*I restriction sites. **(B): **Product from a typical Qβ replicase amplification of RGS mRNA. Lane 1 represents the amount of RGS single-stranded mRNA (-) template added to the Qβ replicase reaction. Lane 2 shows the Qβ replicase reaction product following amplification for 2 hrs at 37°C. Note that Qβ replicase amplifies both (-) and the newly generated (+) strands of the RNA eventually leading to a dsRNA product.

Developing a vector that would allow for the routine application of Qβ replicase for the amplification of target mRNA that could also subsequently be used directly in ribosome display was not straightforward. As Qβ phage has a double-stranded RNA genome, Qβ replicase has a strong bias for replicating its own genome with both RNA (-) and (+) strands serving as templates [14,15]. To adapt this stringent template preference and allow replication of foreign RNAs, pEGX216, a universal mutagenesis vector (Figure 1A) was constructed based around a small multi-cloning site (MCS) inserted into a modified RQ 135_-1_(-) sequence. The RQ 135_-1_(-) sequence is the result of a spontaneous recombination of *E. coli *23 S RNA and the phage λ origin of replication with the resulting sequence efficiently recognized and amplified by Qβ-replicase [16-18]. The integrated MCS did not appear to perturb the secondary structure required by Qβ-replicase to recognize the RNA template. An upstream T7 RNA polymerase promoter sequence on pEGX216 was used to synthesize RNA suitable for Qβ-replicase.

**Figure 2:**
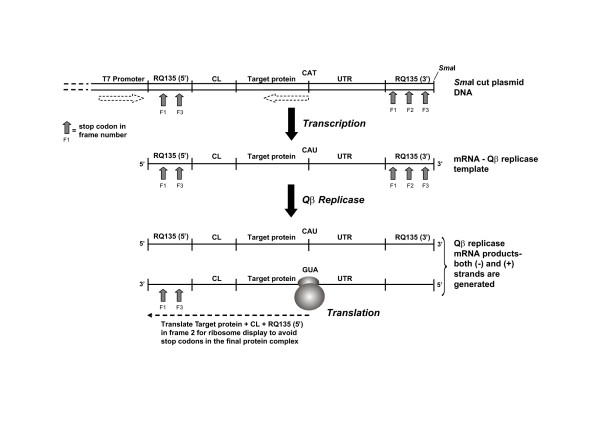
**Coupling Qβ replicase mutagenesis to ribosome display**. The UTR, target gene, and C_L _region are cloned in the reverse orientation (frame 2) relative to the T7 promoter and the RQ 135_-1_(-) sequence to avoid intrinsic stop codons found in the modified RQ 135_-1_(-) sequence. Stop codons are found in frames 1 and 3 of the 5' segment and in all 3 frames of the 3' segment of the RQ 135_-1_(-) sequence (indicated by grey arrows and the script F1 indicating frame 1 etc.). The absence of stop codons downstream from the translational start signal is an essential requirement of ribosome display. The pathway is as follows; the *Sma*I digested plasmid is used as a template for transcribing mRNA via the T7 promoter sequence. Although the UTR-target gene-C_L _message is in the reverse orientation and not suitable for translation, the RQ 135_-1_(-) sequence is in the correct orientation for efficient recognition by Qβ replicase. The resulting mRNA becomes the template for Qβ replicase. Amplification of this mRNA template with Qβ replicase generates both (-) and (+) mRNA. The mRNA in the correct orientation for translation is coupled to ribosome display to produce a protein complex (target gene + C_L _+ RQ 135-5') that remains tethered to the ribosome due to the absence of a stop codon.

For the coupling of mutagenesis to ribosome display, pEGX216 was modified to generate a dual purpose mutagenesis/ribosome display vector (pEGX253) by adding various elements required for efficient translation and for tethering the mRNA to the ribosome complex (see methods and Figure 1A). The mRNA transcribed from this vector was subsequently mutated with Qβ replicase, heat denatured and shunted directly into ribosome display. Note that the 5'-UTR, the Kozak sequence, the target gene, and the C_L _region were cloned in the reverse orientation relative to the T7 promoter to avoid intrinsic stop codons found in our modified RQ 135_-1_(-) sequence. A prerequisite for ribosome display is that there are no stop codons downstream of the translational start signal. Consequently, the target gene sequence, the CL tether, and the downstream segment of our modified RQ sequence (which also forms part of the final tether) could not include a translational stop codon. However, stop codons are found in all three frames of the 3' segment of the RQ 135_-1_(-) sequence but in only two frames of the 5' segment. By cloning into the appropriate frame in the reverse orientation relative to the RQ sequence, stop codons were avoided down stream of the translational start codon. Qβ replicase amplifies RNA in both directions, using both the (+) and (-) RNA strands as templates, consequently, mRNA in the correct orientation for translation and ribosome display was innately generated following Qβ replicase mutagenesis (Figure 2). Note that due to the replication of both (+) and (-) RNA strands, replication of the starting template would be expected to be limited to one round of replication that would stall when inert RNA duplexes are eventually formed by annealed (+) and (-) strands. However, the RQ 135_-1_(-) sequence contains highly developed secondary structures that have been postulated to prevent complementary (+) and (-) strands of RNA from annealing to each other during replication leading to efficient and very rapid replication [19,20]. This property also appears to be important with respect to translation. Ugarov and collegues [17] found that the expression of mRNAs in cell-free translation systems was greatly enhanced as a result of their insertion into RQ135 RNA, again partly attributed to the very stable tertiary structure of the RQ135 RNA. We have found that Qβ replicase amplified mRNA, upon heat denaturing (see materials and methods), was a suitable template for translation without the need to further purify and isolate the correct strand for translation.

### The mutational spectrum of Qβ replicase

**Figure 3:**
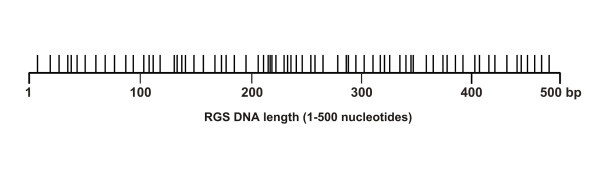
**Distribution of point mutations along the 500 bp RGS**. The spread of Qβ replicase generated mutations along the length of the RGS indicated that Qβ replicase substituted bases randomly across the full length of the RGS mRNA template with no obvious mutational hot-spots. A total of 41,280 bases were sequenced and all point mutations identified were plotted.

**Figure 4:**
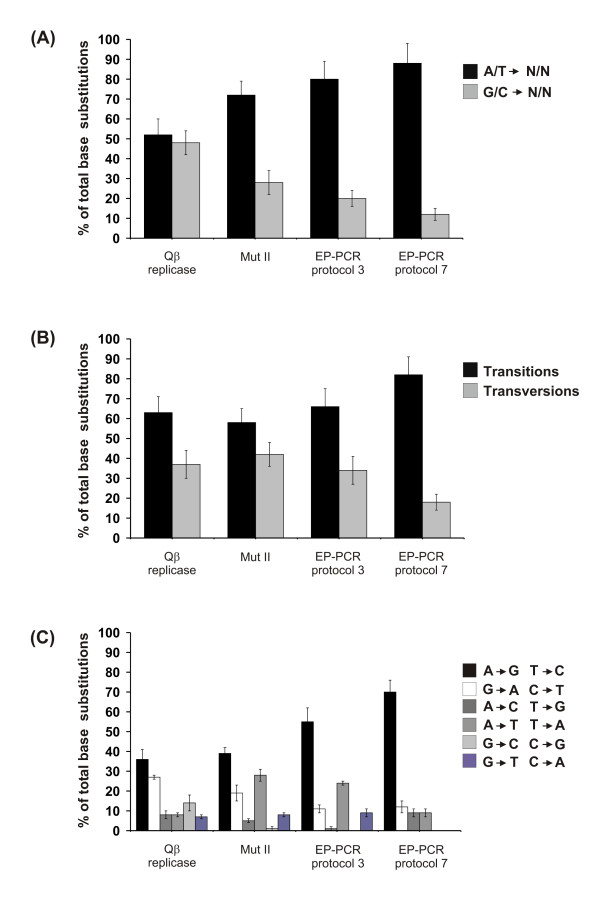
**The mutational spectrum of Qβ replicase, Mut II, and EP-PCR**. **(A): **Qβ replicase did not have significant bias for A/T->N/N (black bars) or G/C->N/N (grey bars) changes. Mut II and EP-PCR using either Mn^2+ ^(manufacture's protocol 3) or Mn^2+ ^with an unbalanced dGTP concentration (manufacture's protocol 7) demonstrated significant A/T->N/N bias. **(B): **The different transition/transversion ratios between the methods. Qβ replicase and Mut II did not show a significant bias for transitions (black bars) over transversions (grey bars) compared to both versions of EP-PCR, which showed a significant preference for transitions. **(C): **The percentage of all possible base substitutions for each of the mutagenesis methods analyzed. All possible base substitutions were recovered with Qβ replicase. The G/C -> C/G transversion was only recovered once with Mut II, which also over represented A/T-> T/A transversions. With EP-PCR (manufacture's protocol 3) A/T-> T/A transversions were over represented, the A/T->C/G transversion was only recovered once and the G/C -> C/G transversion was not recovered. G/C -> C/G and G/C->T/A transversions were not recovered with EP-PCR (manufacture's protocol 7). Both versions of EP-PCR over represented A/T->G/C transitions. Experiments were repeated 2 times with the standard deviation between experiments shown as error bars. The data set used to generate Figure 3 was as follows: 71 point mutations were characterized for Qβ replicase (52,327 bases were sequenced in total with an average mutation rate of 1 point mutation every 737 bases giving a mutation frequency of 1.35). 48 point mutations were characterized for Mut II (6720 bases were sequenced in total with a mutation rate of 1/140 bases giving a mutation frequency of 7.14). EP-PCR manufacture's protocol 3; 74 point mutations were characterized (36,960 bases were sequenced in total with a mutation rate of 1/499 bases and a mutation frequency of 2.00) and manufacture's protocol 7; 43 point mutations were characterized (9,600 bases were sequenced in total with a mutation rate of 1/225 bases giving a mutation frequency of 4.48).

A 500 base pair random gene sequence (RGS) from *Escherichia coli *was used as a model template to establish the mutational spectrum of Qβ replicase and to compare this with the mutational profiles generated by other mutagenesis methods. The 500 bp RDS had a GC content of 53% and did not contain any unusually long stretches of any particular nucleotide. The RGS was embedded into the RQ 135_-1_(-) sequence via the MCS of pEGX216. The mRNA synthesized from this template was subsequently amplified and mutated with Qβ replicase (Figure 1B). The mutated mRNA was converted to cDNA using RT-PCR and cloned. Random clones were selected and sequenced. Under the conditions outlined here, the Qβ replicase error rate was approximately 1 substitution in every 700 bases. The data in Figure 3 demonstrates that Qβ replicase substituted bases randomly across the target template with no obvious hot spots or clustering around particular sequences. Qβ replicase showed an equal preference for inserting A/T or G/C changes, with minimal bias for transitions over transversions (Figure 4A, 4B).

The background error rate (i.e. the mutation rate without Qβ replicase) was measured by sequencing 24,000 base-pairs from the control reaction. Only a single point mutation was detected which was consistent with the predicted error rate expected from a combination of T*aq *DNA polymerase (the highest error rate documented for T*aq *DNA polymerase in the literature is in the order of ~2 × 10^-4 ^[8]) and the error rate contributed by reverse transcriptase (Superscript III™ reverse transcriptase error rate is 3.4 × 10^-5 ^[21]). Together, T*aq *DNA polymerase and Superscript III™ did not significantly contribute to the observed mutation rate seen with Qβ replicase which in this example was in the order of ~7 × 10^-2^.

The mutational spectrum of Qβ replicase compiled on the RGS was directly compared with three DNA-based protocols traditionally used to randomly mutate DNA and generate variant libraries. Two common protocols of EP-PCR (with and without unbalanced concentrations of dGTP) and Mutazyme^R ^II DNA polymerase (Mut II), a recent blend of two different error-prone polymerases that is claimed to produce an even, non-biased spread of mutations (Stratagene), were used to mutate the RGS as outlined above. Our analysis showed that EP-PCR exhibited a typical and well documented bias for A/T over G/C changes and a strong preference for transitions over transversions [7,8]. Both EP-PCR protocols gave similar mutation patterns. Mut II showed a somewhat reduced preference for transitions over transversions but still strongly favored A/T changes over G/C changes (Figure 4A, 4B).

Figure 4C further dissects the data into the individual base substitutions generated with each of the methods. All possible base substitutions were recovered with Qβ replicase and the frequency of all possible transversion substitutions were generally evenly spread. Mut II tended to favor A/T to T/A transversions with the G/C -> C/G transversion recovered only once within the data set. EP-PCR failed to generate many of the possible transversions and showed a distinct preference for A or T changes. All of the methods tended to favor A/T to G/C transitions over G/C to A/T transitions, with EP-PCR showing the greatest bias and Qβ replicase showing the least bias.

### Using RNA-based mutagenesis to engineer the specificity of TEM-1 β-lactamase

As an example of the efficacy of Qβ replicase for generating diverse mRNA libraries, and to provide a direct comparison with published reports of protein evolution using other mutagenesis methods, Qβ replicase mutagenesis was used to modify the well-characterized TEM-1 β-lactamase antibiotic resistance protein of *E. coli*. Although TEM-1 β-lactamase has broad substrate specificity [22], it can not efficiently hydrolyze the extended-spectrum, third generation cephalosporin, cefotaxime [23]. For instance, when wild-type β-lactamase is expressed in *E. coli *from the plasmid pUC19, the minimum inhibitory concentration (MIC) for cefotaxime is 0.02 ug/ml. To modify the ability of β-lactamase to hydrolyze cefotaxime, the protein coding sequence was cloned from the plasmid pUC19 into pEGX216, transcribed to synthesize mRNA, and the mRNA was then mutated with Qβ replicase. The mutated mRNA was converted to cDNA using reverse transcriptase, cloned into a modified pUC19 plasmid (from which the wild-type TEM-1 gene had previously been deleted downstream of the original pUC19 wild-type TEM-1 promoter sequence) and transformed into *E. coli*. Clones were selected for growth on agar plates containing increasing concentrations of cefotaxime.

Resistant colonies chosen after the first round of mutagenesis and selection had a MIC of 20 ug/ml (i.e. growth on plates containing 20 ug/ml cefotaxime) indicating a 1000-fold increase in cefotaxime resistance. Ten clones in total were sequenced yielding five variants. All clones carried the G238S amino acid substitution with 2 clones showing no other mutations. Five clones carried two mutations; E104K and G238S (both G->A transitions). The remaining 3 clones carried the G238S mutation in combination with either F22S (A->G), H153R (A->G) or S267G (T->C). These five variants were used as the basis for the next round of mutagenesis and selection. The mRNA that was generated from each of the variants was mixed together in equal proportions and taken through a second round of mutagenesis and selection as outlined previously with round one. Resistant clones from the second round had a MIC of at least 200 ug/ml (the highest concentration of cefotaxime tested) constituting a minimum 10,000-fold increase in cefotaxime resistance. Again, ten clones were sequenced with all clones carrying in combination with the round 1 mutations E104K and G238S, an extra mutation, M182T (T->C transition) with no silent or other mutations. These three mutations have been described in previous studies of β-lactamase evolution [23]. Interesting to note that two of the key amino acid mutations resulted from G->A substitutions which would be well represented in a Qβ replicase generated library (~30% of total mutations) and less frequent with error-prone PCR (~11% of the total mutations) or Mut II (~20% of the total mutations).

Note that control experiments performed with mRNA treated in an identical fashion to that described above, however, not mutated with Qβ replicase, did not yield any cefotaxime resistant variants at the concentrations of cefotaxime used for selection.

### Coupling RNA-based mutagenesis to ribosome display

Qβ replicase-generated mRNA libraries were directly coupled to ribosome display and used to successfully affinity mature a number of small protein ligands (a single example is presented here with the details of the others to be published elsewhere). Described here is the affinity optimization of a V_NAR _that is based on the antibody-like Ig new antigen receptor unique to sharks [24]. These single domain antibody-like fragments have been reported to bind to their targets via a single, long, finger like loop (analogous to CDR3 of antibodies). A V_NAR _II family member, 12Y-2, originally isolated by Nuttall and coworkers [25], binds to AMA-1, a single, trans-membrane domain protein that is thought to be essential for binding and penetration of the *Plasmodium falciparum *(malaria) parasite (merozoite) into red blood cells [26]. Peptides that bind to AMA-1 have been shown to prevent merozoite invasion [27] suggesting that AMA-1 binders such as 12Y-2 may be biologically useful tools. 12Y-2, however, has only a modest affinity for AMA-1 in the order of 358 nM (K_D_) making it an ideal candidate for affinity maturation.

The protein-coding sequence of 12Y-2 was cloned into the mutagenesis/ribosome display plasmid pEGX253, transcribed to synthesize mRNA, and the mRNA subsequently mutated with Qβ replicase. The mutated mRNA was used directly in ribosome display. One round of selection via ribosome display was performed; the mRNA was recovered and cloned into an expression vector and transformed into *E. coli*. A total of 200 clones were analyzed for binding to AMA-1 by ELISA. A range of variants with affinity increases in the order of 3–7-fold were recovered. One of these clones contained a single mutation K61R (T->A transversion) mapping to framework region 3. This variant was subjected to a second round of Qβ replicase mutagenesis and selection via ribosome display, and again, a total of 200 round 2 clones were analyzed. The best variant identified after this second round (clone 1A-13) had a 22-fold increase in binding affinity (a K_D _of 16 nM) compared with the 12Y-2 parent molecule with an approximate 2-fold increase in the association rate and a 13-fold increase in the dissociation rate [28]). 1A-13 contained two mutations; the parent K61R mutation (isolated in the first round) and P90L (resulting from a C->T transition) generated in the second round. The P90L mutation has also been recently described by Nuttall and co-workers [25] and maps to the CDR3-like region. Western blot analysis and gel filtration chromatography indicated that the expression and monomeric state of 1A-13 appeared unchanged from the starting 12Y-2 parent (data not shown).

## Discussion

In this report, we have outlined an RNA mutagenesis method that was specifically developed to provide an improved approach for the *in vitro *evolution of proteins, in particular, as an effective tool that can be coupled to ribosome display. The RNA-based method is as simple and convenient as PCR to perform and generates a close to ideal random mutational spectrum. Further, Qβ replicase is not only error prone, but also highly processive and productive [29] with reactions containing 1 ng of recombinant template mRNA yielding over 1 μg of double-stranded RNA product in 30 minutes. Consequently, amplification and mutation can be achieved rapidly in a single step.

As a practical demonstration of the effectiveness of the mRNA libraries generated with Qβ replicase, we used the method to alter the substrate specificity of β-lactamase. Several other groups have evolved β-lactamase using a variety of mutagenesis approaches in an attempt to increase the ability of the enzyme to hydrolyze cefotaxime. These studies serve as a solid basis for the evaluation of the data presented here. A comparison is reasonably straight forward as the downstream selection for improved cefotaxime resistance is based on a simple functional assay for growth on an agar plate in the presence of increasing concentrations of cefotaxime, which is expected to be equivalent between laboratories.

As noted above, the cefotaxime MIC for *E. coli *carrying a wild-type β-lactamase plasmid is typically around 0.02 ug/ml. In one of the early examples of β-lactamase protein engineering, Palzkill & Botstein [23] used cassette mutagenesis to isolate variants with a maximum MIC of 0.64 ug/ml. Later, Stemmer [30] used three rounds of DNA shuffling and 2 rounds of back-crossing to yield a TEM-1 variant with an MIC of 640 ug/ml. This cefotaxime resistant mutant contained six amino acid changes (including E104K, G238S and M182T) and an engineered promoter mutation located between the -35 and -10 sites of the β-lactamase P3 promoter that increased β-lactamase expression levels by 2–3-fold. Finally, Zaccolo and Gherardi [31] isolated TEM-1β-lactamase variants with activity against cefotaxime that was reported to be 20,000-fold higher (~400 ug/ml) than wild-type TEM-1 by screening small pools of hyper-mutated clones (<1.5 × 10^5^) from libraries containing up to 27 nucleotide substitutions per gene. Zaccolo and Gherardi [31] required 3 rounds of mutagenesis and selection to isolate clones containing the mutations E104K, G238S and M182T, 2 silent mutations, and a mutation in the pBR322 promoter region. We presume that our second round clones isolated in this study were comparable to the highly resistant Zaccolo and Gherardi clone since the same amino acid substitutions were selected in both studies (notwithstanding the promoter mutation and the copy number difference between the pBR322 plasmid used by Zaccolo and Gherardi and the pUC19 plasmid used in this study). Interesting to note that the unselected TEM-1 libraries of Zaccolo and Gherardi contained a mean mutation frequency of 8.2 and 27.2 substitutions per gene length, however, clones selected on the basis of cefotaxine resisitance showed only 1–11 mutations at the DNA level. It is clear that although increasing the mutation rate allowed a greater diversity to be sampled, it was from the sequences that carried the least number of mutations that functional variants were isolated [10].

This report highlights the efficiency of the Qβ replicase RNA methodology. Comparable cefotaxime resistant variants were selected relatively quickly (only two rounds of mutagenesis and selection) and with no silent or other superfluous mutations, an approach that was accomplished through what we have termed a "minimal mutational pathway". This approach is based on the premise that a small number of key amino acid substitutions at crucial positions can have a dramatic effect on the properties of the protein. This type of strategy can only be successfully applied when a low, unbiased mutation frequency is used to generate a large number of extremely diverse variants that allow for complete sampling of the immediate sequence space neighborhood. Moore and Maranas [9] noted that base substitution bias introduced by *Taq *DNA polymerase under error-prone conditions would render some variants more likely than others, effectively reducing the overall library diversity. Patrick and coworkers hypothesised [10] that given that directed evolution is most likely to identify an improved variant when a library is maximally diverse, this would certainly be the case when all variants in a library are equally probable.

The minimal mutational pathway also provides a number of other significant advantages. Due to the relatively low mutation rate, variants have few, if any superfluous or silent base changes, ensuring that a significant fraction of the variant pool being screened is functional. By minimizing the introduction of unnecessary amino acid changes, the method also avoids collateral damage to proteins. Unnecessary amino acid substitutions can have significant negative effects on a target protein including increased immunogenicity [32-34], a reduction in expression level and protein stability [35], amongst others. In particular, altering the immunogenicity status of a protein can have a significant downstream impact on the effectiveness of a product as a potential therapeutic [36].

Without doubt, one of the significant advantages of a RNA-based mutagenesis strategy is the potential to convey variant mRNA libraries directly into selection approaches such as ribosome display with little intervention, therefore maintaining maximum library diversity during the selection process. The effectiveness of Qβ replicase-generated mRNA libraries coupled to ribosome display was demonstrated by affinity maturing 12Y-2. Two discrete rounds of Qβ replicase mutagenesis and ribosome display yielded the 1A-13 mutant that showed a 22-fold increase in binding to AMA-1 relative to the 12Y-2 parent molecule. 12Y-2 has also been affinity-matured by Nuttall and colleagues [25] using EP-PCR and 3 rounds of selection via phage display. This group isolated several mutants of 12Y-2 with the best of these variants carrying the P90L mutation and showing an 8-fold enhanced affinity for AMA-1.

Apart from demonstrating the relative efficiency of the Qβ replicase mutagenesis approach, the 1A-13 example is also highlighted here to illustrate the potential difficulties that challenge directed antibody or protein engineering strategies that focus mutagenesis on predicted target contact regions alone. The structure of 12Y-2 has recently been published and the authors suggest that the binding of 12Y-2 is essentially mediated via the relatively long CDR3-equivalent loop [37,38]. While amino acid 90 falls within the expected attachment region in this loop, the K61R mutation is situated along the exposed flank of the V_NAR_, apparently outside the antigen-binding paratope. Our finding suggests that the K61R mutation may either rotate or flex CDR3 and improve the interaction of CDR3 with AMA-1 or that this distal exposed site may in fact have a significant functional role in the binding interaction by making contact with peripheral remote locations on AMA-1 relative to the epitope targeted by CDR3 [28]. Potentially, the K61R mutation may fall within an additional hyper-variable region that forms a belt around the periphery of the molecule [39]. This additional hyper-variable region may be an important consideration when engineering the affinity of IgNARs in general. Certainly, a directed protein engineering approach exclusively targeting the extended CDR3 loop would not have identified the 1A-13 variant.

## Conclusion

The success of the minimal mutation pathway approach to protein optimization presented here may be pertinent in light of the debate in the literature over the benefits of exploiting large random libraries created at a low mutation frequency versus smaller libraries manufactured using saturation mutagenesis [31]. Our data suggests that the nature and diversity of mutations generated within a gene library governs the outcome, and not the mutation frequency *per se- *the wider the spectrum of single amino acid variations that are generated along the gene length (achieved through unbiased nucleotide substitutions), which can then be sampled from a library, the more likely that the best possible amino acid changes required for improving protein properties will be identified. The practical implications of our results are that suitable improved protein candidates generated through *in vitro *protein optimization technologies can be selected using significantly fewer rounds of mutagenesis and selection, and with little or no collateral damage to the protein or its mRNA.

## Methods

### Plasmid construction

A multi-cloning site comprising of *Xho*I, *Sac*II, *Not*I, *Nco*I, *Sfi*I and *Xho*I was inserted into a highly modified RQ 135_-1_(-) sequence [16,17] essentially dividing RQ 135_-1_(-) into a 5' end and a 3' end with the multi-cloning site buried in the middle of the sequence. PCR was used to add a *Hind*III site and T7 promoter region to the 5' end, and a *SmaI *site to the 3' terminus of the RQ 135_-1_(-) sequence. The construct was inserted into the *Hind*III and *Sma*I sites of pUC18 (New England Biolabs). This plasmid was designated pEGX216. The following elements were added to pEGX216 to construct pEGX253, which was used to generate mRNA for Qβ replicase mutagenesis and subsequent ribosome display. A 510 base-pair 5'-UTR followed by a complete Kozak sequence was added via the *SfiI *and *NcoI *sites; the target gene (in this case, the 12Y-2 coding sequence) was inserted between the *NcoI *(also serving as the start codon) and *NotI *sites; and directly downstream of the target gene (between the *NotI *and *SacII *sites) was inserted a 316 bp fragment of the mouse antibody constant light chain (C_L_) devoid of stop codons that served to link the newly synthesized protein to the ribosome complex during ribosome display.

### Generating RNA template suitable for Qβ replicase

To generate an mRNA template suitable for Qβ replicase, plasmids (either pEGX216 or pEGX253) were linearized with *SmaI *to generate a blunt end fragment that terminates with CCC at the 3' end of the RQ 135_-1_(-) sequence. As T7 polymerase initiates transcription with GGG, the resulting mRNA transcripts contained both the 5'-GGG and 3'-CCC termini thought to be essential elements for template recognition by Qβ-replicase [19]. T7 polymerase often inserts a terminal A nucleotide to the 3' CCC sequence, however, this did not appear to significantly influence RNA template recognition. Run off transcription on the linear DNA template was performed by adding 40 mM Tris-HCl (pH 7.9), 6 mM MgCl_2_, 2 mM spermidine, 10 mM dithiothreitol, 1 mM each of rCTP, rUTP, rGTP, and rATP, 2 U RNase inhibitor (Promega), 20 U T7 polymerase (Promega) to 200 ng DNA template and incubating at 37°C overnight. RNA was DNase-treated to remove the DNA template (RQ1 DNase, Promega) and purified (RNeasy, Qiagen) prior to amplification with Qβ replicase.

### Qβ replicase reaction

100 ng of mRNA was pre-heated for 2 min at 95°C in a thermocycler and permitted to cool slowly to room temperature. The mRNA was mixed with 40 mM Tris-HCl (pH 7.9), 21 mM MgCl_2_, 2 mM spermidine, 10 mM dithiothreitol, 1 mM each of rCTP, rUTP, rGTP, and rATP, 2 U RNase inhibitor (Promega) and 200 nM Qβ replicase (prepared in house following the method of Moody *et al*. [40]) and incubated for a minimum of 120 min at 37°C. Qβ replicase products were visualized by combining 5 ul of the Qβ replicase reaction with loading buffer (Invitrogen), and separated via elecrophoresis on 1% agarose gels containing ethidium bromide. The remaining mRNA was purified (RNeasy; Qiagen) to remove excess MgCl_2_prior to ribosome display.

### Determining mutation rates

The mutation frequency and nucleotide bias of Qβ replicase was measured using mRNA transcribed from the RGS DNA which was imbedded into the RQ 135_-1_(-) sequence of pEGX216 using the X*ho*I restriction sites. The plasmid was linearized with *SmaI *and was used in a transcription reaction to generate mRNA template suitable for Qβ replicase. The mRNA template was amplified with Qβ replicase and subsequently reverse transcribed and PCR-amplified (Superscript III™ RT-PCR; Invitrogen) prior to blunt-end cloning into pPCR-Script Amp SK (+) (Stratagene), transformed into *E. coli *strain HB2151, and clones chosen at random were sequenced. The control reaction was processed as outlined above, however, the mRNA was not amplified with Qβ replicase.

Error-prone PCR (Diversify PCR Random Mutagenesis Kit; Clontech Laboratories) using the manufacture's protocol 3 (containing 320 uM Mn^2+ ^and 40 uM dGTP) and protocol 7 (containing 640 uM Mn^2+ ^and an unbalanced dGTP concentration of 120 uM) and Mut II (GeneMorph II Random Mutagenesis Kit; Stratagene) were used to mutate RGS DNA closely following the protocols outlined by the manufacturers. The total amount of target template for each reaction was 1 ng with reactions adjusted to give approximately a 1000-fold amplification (total yield/temple amount). Mutated DNA was subsequently cloned into pPCR-Script Amp SK(+), and as above, transformed into *E. coli*, and random clones sequenced. Since it was not possible to differentiate which of the nucleotide pair was misincorportated during Qβ replicase amplification and subsequent RT-PCR, all possible nucleotide substitutions were grouped into six complementary categories. The mutation rate was measured from a total of three replicate experiments for each method. The data is presented as mutation frequency (number of nucleotide substitutions per kb), with a minimum of 40 base substitutions characterized for each method.

### Evolution of β-lactamase

The TEM-1 β-lactamase gene was cloned into pEGX216 via the *XhoI *restriction sites, mRNA was transcribed via the T7 promoter, and the mRNA subsequently mutated with Qβ replicase. One ng (approximately 10^11 ^molecules) of mutated mRNA was converted to cDNA and amplified using high fidelity RT-PCR and gene specific primers. The entire RT-PCR reaction was purified, restricted with *NcoI *and *NotI *and ligated into 25 ng of a modified pUC19 vector (New England Biolabs) to replace the wild-type TEM-1 gene. The modified pUC19 vector was constructed by generating *NcoI *and *NotI *sites at the terminal ends of the pUC19 wild-type TEM-1 gene sequence by site directed mutagenesis (QuickChange Mutagenesis) to allow the wild-type TEM-1 gene to be deleted (leaving the original upstream regulating sequences intact). 10 ng of the cloned DNA was used to transform *E. coli *XL10-Gold cells (Stratagene) with an estimated transformation efficiency 5 × 10^9 ^colonies per ug pUC19 DNA. The entire transformation mix (potentially representing a library of approximately 10^7 ^variants) was plated onto solid nutrient media containing cefotaxime (Sigma) concentrations of either 5, 10 or 20 ug/ml. As cefotaxime resistance on solid media is cell-density-dependant, colony numbers were standardized to 300–500 colonies per plate and grown at 37°C for 30 h. Plasmid DNA from resistant clones that grew on the cefotaxime supplemented media was extracted and sequenced. The DNA from the best clones from the first round of mutagenesis and selection were taken through a second round of mutagenesis and selection by digesting the plasmid DNA of the individual clones with *NcoI *and *NotI *and ligating back into pEGX216. The plasmids were then mixed in equal proportions to repeat the mutagenesis and selection process. Second round variants were selected on solid nutrient media containing cefotaxime concentrations of either 50, 100 or 200 ug/ml. A control experiment run in parallel was identical with the omission of the Qβ replicase mutagenesis step.

### Ribosome display

Ribosome display was performed by adding 2 ug of heat denatured Qβ replicase mutated mRNA (heated to 75°C for 2 min and then cooled rapidly to 4°C) to a 50 ul rabbit reticulocyte based translation reaction (Flexi rabbit reticulocyte lysate system; Promega) in an RNase-free microfuge tube (Ambion) following the manufacturer's recommendations and allowing translation to proceed for 25 min at 30°C before diluting the translation mix with the addition of RNase-free, biotin-free skim milk (4% w/v), PBS, and 5 mM MgCl_2 _[11,12,41]. 1 nM biotinylated AMA-1 (biotinylated with EZ-Link™ Sulfo-NHS-LC-Biotin; Pierce) was added directly to the diluted translation mix in the microfuge tube and rocked on ice for 5 h prior to the addition of 2000 nM AMA-1. The mix was rocked on ice for a further 2 h. Ribosome complexes that remained attached to the biotinylated AMA-1 were recovered with streptavidin coated magnetic beads (Dynal). Beads were subsequently washed three times each with PBS containing 5 mM MgCl_2 _and 0.01% Tween 20 and two times with PBS containing 5 mM MgCl_2 _before being re-suspended in 40 ul dH_2_O that had been pre-heated to 65°C to disrupt the ribosome complexes. The beads were removed and the supernatant was used directly in RT-PCR to recover mRNA using vector specific primers. The RT-PCR product was gel purified (Qiagen), digested with *NcoI *and *NotI *and ligated into pGC FLAG/HIS for expression and analysis [42]. The DNA from designated round 1clones that were to be taken into a second round of mutagenesis and selection were ligated into pEGX253 and processed as outlined above with round 1. Second round panning incorporated increased selection pressure by increasing the incubation phase to 16 h prior to mRNA recovery. Following the second round of mutagenesis and panning, 500 individual clones were again analysed. Bacterial periplasmic expression and analysis (ELISA and biosensor binding) of 12Y-2 clones were as outlined previously [23].

## Authors' contributions

GK designed the study, developed the RNA mutagenesis assay, developed the vectors, directed the research group and drafted the manuscript. ELS determined the mutagenesis spectrum of all the mutagenesis methods analysed, AR, RKC, LP-B and GK performed the ribosome display experiments, MAS performed the ELISA assays used to screen the clones originating from ribosome display and the protein expressions and purification of candidate proteins, ASR performed the BIAcore analysis and also protein expressions and purifications, ND and GC synthesized and purified Qβ replicase, and GC designed the study, helped to draft the manuscript, and also guided the project. All authors read and approved the final manuscript.
